# Chromosome-Scale Genome Architecture and Historical Demography of the Southern White Rhinoceros

**DOI:** 10.3390/biology15120924

**Published:** 2026-06-12

**Authors:** Jiong Zhou, Xiaofang Zhou, Fenglei Zhang, Wu Chen, Lei Chen

**Affiliations:** 1 Shaanxi Key Laboratory of Qinling Ecological Intelligent Monitoring and Protection, School of Life Sciences and Technology, Northwestern Polytechnical University, Xi’an 710072, China; 2 Guangzhou Wildlife Research Center, Guangzhou Zoo, Guangzhou 510070, China

**Keywords:** *Ceratotherium simum simum*, genome assembly, comparative genomics, structural variation, major histocompatibility complex, demographic history, conservation genomics

## Abstract

The white rhinoceros (*Ceratotherium simum*) offers a unique model for investigating the genomic consequences of extreme demographic bottlenecks. However, the fragmented southern white rhinoceros genome assembly has limited chromosome-scale structural and evolutionary comparisons with the functionally extinct northern subspecies. Here, we report a chromosome-scale genome assembly for the southern white rhinoceros by integrating Oxford Nanopore Technology long-read sequencing, Illumina short-read polishing and high-throughput chromosome conformation capture (Hi-C) scaffolding. The final assembly spans 2.48 Gb and achieves a contig N50 of 42.06 Mb, representing a 452-fold improvement in contiguity over the previous assembly. In total, 2.46 Gb of sequence was anchored to 40 autosomes plus the X and Y chromosomes. Genome annotation identified 1.13 Gb of repetitive elements (45.7% of the assembly), 22,593 protein-coding genes, and 100.68 Mb of segmental duplications. Inspection of the major histocompatibility complex class II gene region further supported the local assembly and annotation reliability, revealing conserved gene composition and order between the southern and northern white rhinoceroses. Whole-genome comparison with the northern white rhinoceros assembly indicated extensive chromosome-scale synteny, along with localized structural variants between the two subspecies, including 111 inversions spanning 33.48 Mb and 497 translocations spanning 36.48 Mb. Furthermore, coalescent demographic reconstruction indicated asynchronous Pleistocene population dynamics for southern and northern white rhinoceroses, reflecting divergent responses to historical climate oscillations. Both subspecies also exhibit lower recent effective population sizes than estimated Pleistocene ancestral levels, underscoring persistent conservation concern. This assembly provides a useful resource for evaluating the genomic consequences of historical bottlenecks, informing future genomic-rescue plans, and strengthening the comparative framework for rhinoceros conservation and evolutionary genomics.

## Simple Summary

The white rhinoceros (*Ceratotherium simum*) includes two closely related subspecies with very different conservation histories. The southern white rhinoceros has recovered from a severe historical bottleneck, whereas the northern white rhinoceros is now functionally extinct. In this study, we generated a high-quality chromosome-level genome assembly for the southern white rhinoceros. This new genome improves the available genomic resources and supports chromosome-scale comparisons with the northern white rhinoceros. We found that the two subspecies are largely similar at the chromosome scale but show localized structural differences. We also examined an important immune-related genomic region and reconstructed their historical population changes, indicating different demographic trajectories during the Pleistocene. This genome provides a valuable resource for future studies of rhinoceros evolution, population history, and conservation.

## 1. Introduction

The family Rhinocerotidae comprises five extant species distributed in Africa and Asia, and their conservation status ranges from Near Threatened to Critically Endangered according to the International Union for Conservation of Nature (IUCN) Red List and taxonomic assessments [1,2,3]. The white rhinoceros (*Ceratotherium simum*) is one of the largest terrestrial herbivores and is a megagrazer that feeds predominantly on grasses in African grassland and savanna habitats [4,5,6]. The species is generally divided into two subspecies, the southern white rhinoceros (*Ceratotherium simum simum*; *C. s. simum*) and the northern white rhinoceros (*Ceratotherium simum cottoni*; *C. s. cottoni*), which have experienced contrasting recent conservation histories [7,8,9]. The southern white rhinoceros recovered from a severe historical bottleneck of fewer than approximately 50–100 individuals in the early twentieth century to more than 20,000 individuals by the early twenty-first century, although this recovery remains conservation-dependent [1,9,10]. In contrast, the northern white rhinoceros is functionally extinct, with only two non-reproductive captive females remaining [8]. This contrast provides a useful system for investigating how severe demographic bottlenecks shape genome-wide diversity, inbreeding, recovery potential, and long-term population viability [7,9,11,12].

High-quality reference genomes are important for resolving complex evolutionary histories and for enabling conservation genomic analyses of demography, inbreeding, adaptive variation, and genome architecture [13,14,15]. Previous population genomic studies have revealed declining heterozygosity and elevated inbreeding in both white rhinoceros subspecies [7], and phylogenomic analyses have resolved deep relationships across Rhinocerotidae [2]. However, precise chromosome-scale structural comparisons between the two white rhinoceros subspecies remain limited by assembly contiguity [15,16]. The recent chromosome-level assembly of the northern white rhinoceros demonstrated the value of structurally resolved reference genomes for comparative and conservation applications [8]. Compared with this need, the previous southern white rhinoceros reference genome, CerSimSim1.0 (GCA_000283155.1), was highly fragmented, which limited integrated analyses of repetitive regions, segmental duplications, and large structural variants. Such fragmented assemblies can obscure complex genome architecture and reduce the reliability of structural variant detection, particularly in repeat-rich regions [15,16].

Here, we report a chromosome-level genome assembly of the southern white rhinoceros, generated by integrating Oxford Nanopore Technologies (ONT) long-read sequencing, Illumina short-read polishing, and high-throughput chromosome conformation capture (Hi-C) scaffolding. The resulting assembly spans 2.48 Gb with a contig N50 of 42.06 Mb and Benchmarking Universal Single-Copy Orthologs (BUSCO) completeness of 99.72%, anchored across 40 autosomes and both sex chromosomes. Building on this resource, we provide a comprehensive structural annotation of the southern white rhinoceros genome, resolving repetitive landscapes, protein-coding gene content, and segmental duplications. By resolving the major histocompatibility complex class II locus, a key immune region involved in antigen processing and presentation [17], and by performing whole-genome synteny analysis, our findings suggest that white rhinoceros subspecies divergence is associated with localized structural differences rather than large-scale chromosomal rearrangements. Furthermore, we reconstructed historical effective population size trajectories using Pairwise Sequentially Markovian Coalescent (PSMC) for both subspecies and SMC++ for five southern white rhinoceros genomes, indicating asynchronous Pleistocene demographic fluctuations that reflect their independent ecological histories. Together, these analyses provide a chromosome-resolved comparative framework for evaluating how historical bottlenecks are reflected in genome architecture, functional annotation, structural variation, and long-term demographic trajectories in white rhinoceroses.

## 2. Materials and Methods

### 2.1. Sample Preparation and Sequencing

A male southern white rhinoceros from Guangzhou Zoo was used for genome sequencing in this study. This individual is biologically distinct from the specimen utilized for the earlier CerSimSim1.0 short-read assembly (GCA_000283155.1). Whole blood was collected under veterinary supervision and was immediately preserved for genomic DNA extraction. All animal procedures were reviewed and approved by the Animal Ethics Committee of Guangzhou Zoo and Guangzhou Wildlife Research Center under approval number GZZOO20240101H and were conducted in accordance with the guidelines in the Guide for the Care and Use of Laboratory Animals in China.

High-quality genomic DNA was extracted from whole blood using the DNeasy Blood & Tissue Kit (Qiagen, Germantown, MD, USA) according to the manufacturer’s protocol. DNA concentration, purity, and integrity were evaluated using agarose gel electrophoresis and a Qubit fluorometer (Thermo Fisher Scientific, Waltham, MA, USA). For de novo genome assembly, sequencing libraries were prepared for ONT long-read sequencing according to the manufacturer’s instructions and subsequently sequenced on a PromethION platform (Oxford Nanopore Technologies, Oxford, UK). To obtain highly accurate short reads for genome polishing and assembly evaluation, short-read libraries were also constructed and sequenced on the Illumina platform (Illumina, San Diego, CA, USA). In addition, a Hi-C library was prepared from whole-blood cells following a standard in situ Hi-C protocol with minor modifications. Briefly, cross-linked blood cells were lysed, chromatin was digested with MboI, and the resulting sticky ends were filled in with biotinylated nucleotides before proximity ligation. After cross-link reversal and DNA purification, the ligation products were fragmented, biotinylated fragments were enriched, and a paired-end Hi-C library was constructed. The library was sequenced on the Illumina NovaSeq 6000 platform (Illumina, San Diego, CA, USA) to generate 150 bp paired-end Hi-C reads for chromosome-level scaffolding. Library preparation and sequencing for all sequencing libraries were performed by Haorui Gene Technology Co., Ltd. (Xi’an, China).

Raw sequencing data were filtered before downstream analyses. ONT reads shorter than 1 kb were removed before assembly. Illumina short reads were filtered to remove adapter sequences, low-quality bases, and reads with excessive ambiguous bases.

### 2.2. Genome Assembly and Assessment

ONT long reads longer than 1 kb were self-corrected and assembled de novo using NextDenovo v2.2-beta.0 [18]. Draft contigs were subsequently polished using NextPolish v1.2.1 [19] with three rounds of long-read polishing and three rounds of short-read polishing. For Hi-C-based scaffolding, the contig index was first built using SAMtools v1.9 [20]. Hi-C reads were then mapped to the polished contigs using Chromap v0.2.5-r473 [21]. Based on Hi-C interaction signals, contigs were sorted, pruned, and optimized using YaHS v1.2 [22] with default parameters. The Hi-C contact matrix was generated using Juicer v1.6 [23], and candidate chromosome-scale assemblies were manually reviewed and refined in Juicebox v1.6 [24]. Genome completeness was evaluated using BUSCO (Benchmarking Universal Single-Copy Orthologs) v5.1.2 [25] with the mammalia_odb10 dataset [26] and Compleasm v0.2.2 [27], and overall assembly statistics were further summarized using BlobToolKit v4.5.0 [28]. Genome completeness and consensus quality value (QV) were also assessed using Merqury v1.3 [29] based on Illumina short-read k-mers. The QV score indicates confidence in the accuracy of assembly sequences supported by the sequencing data.

### 2.3. Repeat Annotation

Repetitive elements in the southern white rhinoceros genome were identified using a homology-based strategy. Known transposable elements were annotated using RepeatMasker v4.1.6 [30] against the Repbase library v.20181026 [31].

### 2.4. Segmental Duplication Annotation

Segmental duplications (SDs) were identified using BISER v1.4 [32] on the soft-masked genome with the parameters --max-error 20, --max-edit-error 10, and --kmer-size 31. Candidate SDs were filtered using criteria adapted from the T2T-CHM13 segmental-duplication analysis [33], retaining alignments with gap-compressed identity > 90%, gapped sequence proportion ≤ 50%, aligned length > 1 kb, and satellite content ≤ 70% as estimated from RepeatMasker annotations. The filtered SDs were visualized using Circos v0.69-8 [34], and overlaps between SDs and annotated genes were calculated using custom scripts.

### 2.5. Protein-Coding Gene Prediction and Functional Annotation

Protein-coding genes were annotated using the EviAnn (v2.0.5) pipeline [35], which directly integrates multiple lines of evidence to construct consensus gene models. Prior to integration, de novo gene predictions were generated on the soft-masked genome using ANNEVO (v2.0). Additionally, homology-based annotations were transferred from the highly contiguous horse TB-T2T reference (GCF_041296265.1) using LiftOn (v1.0.5) [36] to leverage well-curated perissodactyl models. For transcriptomic and external protein evidence, a public *Ceratotherium simum* RNA-seq dataset (SRR5647986) and homologous protein sequences from representative mammals (horse, tapir, cattle, and human) were utilized. Finally, the ANNEVO predictions, LiftOn annotations, homologous proteins, and RNA-seq FASTQ files were simultaneously input into EviAnn. EviAnn automatically handled the RNA-seq read alignment and transcript assembly internally, synthesizing all provided evidence into the final protein-coding gene set. Predicted proteins were functionally annotated against public databases including Gene Ontology (GO) [37], InterPro [38], Kyoto Encyclopedia of Genes and Genomes (KEGG) [39], Swiss-Prot and TrEMBL [40], and EggNOG [41]. A gene was considered functionally annotated when it had at least one significant hit satisfying a strict E-value threshold of <1 × 10^−5^.

To evaluate annotation quality in a complex immune-associated region, we analyzed the major histocompatibility complex class II (MHC-II) locus in both the southern white rhinoceros and the northern white rhinoceros using horse as the reference. Equine MHC-II gene models from the TB-T2T annotation were transferred independently to the southern and northern white rhinoceros assemblies using LiftOn and subsequently inspected for gene completeness, order, and orientation. GeneWise v2.4.1 [42] was additionally used to validate the structure of representative MHC-II genes and to confirm the consistency of coding sequences and exon–intron boundaries in this region. Comparative evaluation of the MHC-II region was then performed among the two white rhinoceros subspecies, black rhinoceros, horse, and tapir. The equine MHC-II region has been previously resolved by long-read sequencing and detailed manual annotation, making it a suitable benchmark for locus-level validation and cross-species comparison [43]. For black rhinoceros and tapir, the corresponding MHC-II regions were identified from publicly available genome assemblies using the same homology-based annotation and manual inspection strategy.

### 2.6. Phylogenetic Tree Construction and Divergence Time Estimation

To provide an evolutionary framework for the comparative genomic analyses, orthologous gene families were inferred among the southern white rhinoceros, northern white rhinoceros, tapir, horse, cattle, pig, mouse, and human using OrthoFinder v2.5.5 [44]. A total of 11,831 single-copy orthologous protein-coding genes were identified and retained for phylogenetic analysis. For each single-copy ortholog, protein sequences were aligned using MUSCLE v3.8.1551 [45]. The corresponding coding-sequence alignments were generated from the protein alignments using PAL2NAL v14 [46], and fourfold-degenerate (4D) sites were extracted from the codon alignments. The concatenated 4D-site alignment was used to infer the phylogenetic tree using IQ-TREE 3 v3.0.1 [47], with ModelFinder and 1000 ultrafast bootstrap replicates. Divergence time estimation was performed using MCMCTree in PAML v4.9 [48]. Time calibration priors were assigned to all internal nodes based on fossil-calibrated divergence-time estimates obtained from TimeTree (www.timetree.org). The resulting time-calibrated tree was used to confirm the phylogenetic placement of the southern white rhinoceros and to contextualize downstream comparisons of genome structure and demographic history.

### 2.7. Whole-Genome Synteny Analysis

For whole-genome synteny analysis, the chromosome-level assembly of *C. s. simum* was used as the reference and the *C. s. cottoni* assembly was used as the query. Pairwise whole-genome alignment was performed using minimap2 v2.28-r1209 [49], and alignments were sorted and indexed with SAMtools [20]. Structural rearrangements were identified using SyRI v1.7.1 [50] in BAM mode (-F B) and variant categories were summarized from the SyRI output. Genome-wide visualization of syntenic and rearranged blocks was generated using plotsr v1.1.1 [51]. Raw ONT reads were mapped back to the southern white rhinoceros assembly to assess read support for SyRI-predicted structural variants (SVs). For each SV locus, the two reference-side breakpoints were evaluated separately, and an SV was considered ONT-supported only when both breakpoints were independently spanned by ONT reads. The numbers of SVs supported under different read-depth thresholds are summarized in Table S1. Representative large SVs were further inspected in IGV for breakpoint-spanning and orientation-consistent read support (Figure S1).

To identify genes directly associated with structural variation, SyRI-predicted SV coordinates were intersected with gene annotations from the southern white rhinoceros genome in this study. Gene, exon, and coding sequence (CDS) intervals from different transcript isoforms of the same gene were merged to avoid redundant counting. Genes whose gene bodies, exons, or CDS regions overlapped SV intervals were defined as SV-associated genes. For downstream enrichment analysis, genes with CDS regions overlapped by SVs ≥ 10 kb were retained and analyzed using Metascape [52].

### 2.8. Demographic History

Historical changes in effective population size were inferred using PSMC v0.6.5-r67 [53] and SMC++ v1.15.4 [54]. For both analyses, single-nucleotide polymorphism (SNP) discovery and genotype filtering were performed using the same workflow. For PSMC analysis, Illumina short-read data generated in this study were used for the southern white rhinoceros individual, whereas publicly available Illumina short reads of a northern white rhinoceros individual were obtained from the National Center for Biotechnology Information (NCBI) Sequence Read Archive (SRA) under accession number SRR11428438. The southern and northern white rhinoceros individuals were processed independently using the same read filtering, alignment, variant calling, masking, and PSMC parameter settings.

Raw Illumina reads were first filtered using fastp v0.20.1 [55] to remove adapter sequences and low-quality reads. Clean reads were aligned to each reference genome using BWA-MEM in BWA v0.7.17 [56] with read-group information, and the resulting alignments were converted to BAM format, sorted, and indexed using SAMtools v1.9 [20]. Alignments with mapping quality <30 and reads flagged as duplicates were excluded. Variants were called using BCFtools v1.9 [57]. Only biallelic SNPs with site quality ≥30 were retained. X- and Y-linked regions were excluded to avoid sex-linked bias, and only autosomal SNPs were retained for demographic analyses. Genotypes with sequencing depth <8 or >80 were masked as missing before downstream demographic inference. Genome-wide heterozygosity was estimated as the number of heterozygous SNPs divided by the total number of callable autosomal sites. These genotype-state summaries were used to evaluate the completeness of the variant datasets used for demographic inference.

PSMC was run using a refined interval pattern, -p “1+1+1+1+25*2+4+6” [58] and the conventional pattern -p “4+25*2+4+6”. For demographic scaling, both the southern and northern white rhinoceros trajectories were converted using a generation time of 25 years and a neutral mutation rate of 2.2 × 10^−8^ substitutions per site per generation [59]. These values are consistent with parameters commonly used in previous rhinoceros demographic analyses [2]. To assess robustness, 100 bootstrap replicates were performed.

To complement the PSMC results, demographic history was further inferred using SMC++ v1.15.4 [54] based on genome-wide SNP datasets from five southern white rhinoceros individuals. These included the individual sequenced in this study and four additional individuals obtained from the NCBI SRA under accession numbers SRR387388, SRR5852740, SRR5852739, and SRR5852738 (Table S2). The same read filtering, alignment, variant calling, and genotype filtering criteria described above were applied to all individuals. SMC++ was run using the filtered autosomal SNP dataset, and the same generation time and mutation rate were used for demographic scaling.

## 3. Results

### 3.1. Chromosome-Level Genome Assembly of C. s. simum

We generated a chromosome-level genome assembly for the southern white rhinoceros by integrating 195.6 Gb of ONT long-read data (81.5×), 151.8 Gb of Illumina short-read data (63.25×), and 34.3 Gb of Hi-C data (14.29×) (Table S3). The final assembly spans 2.48 Gb, anchoring 2.46 Gb of sequence into 40 autosomes and the X and Y chromosomes. The genome architecture exhibits high continuity, achieving a contig N50 of 42.06 Mb and a scaffold N50 of 66.38 Mb (Table 1).

**Figure 1:**
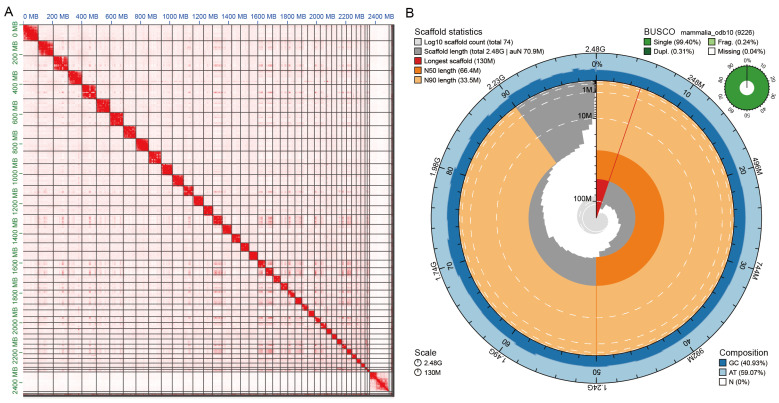
Chromosome-level assembly overview of the southern white rhinoceros genome. (**A**) High-throughput chromosome conformation capture (Hi-C) interaction heatmap of the final assembly, showing strong intrachromosomal contact enrichment and clear chromosome boundaries across the assembled chromosomes, including the X and Y chromosomes. (**B**) BlobToolKit snail plot summarizing the major assembly statistics.

The assembly showed high base accuracy and structural integrity. The consensus quality value (QV) reached 43.52, surpassing the Vertebrate Genomes Project (VGP) standard of QV40, while BUSCO analysis recovered 99.72% complete mammalian orthologs (9200 of 9226 genes in the mammalia_odb10 dataset) (Table 1). Furthermore, the Hi-C contact heatmaps confirmed the accuracy of chromosomal anchoring and orientation, displaying strong diagonal interaction signals with minimal interchromosomal noise (Figure 1A). The BlobToolKit analysis further corroborated the high assembly completeness, strong scaffold continuity, and a stable GC content of 40.93% (Figure 1B). Compared to the previous *C. s. simum* reference genome (GCA_000283155.1), which was generated independently and was not derived from the individual sequenced in the present study, our assembly yields a 452-fold increase in contig N50 (from 93 kb to 42.06 Mb) and reduces the total contig count from 57,823 to 297 (Table S4). This highly contiguous genome provides an unprecedented resource for resolving complex genomic features and structural variation in the white rhinoceros.

### 3.2. Genome Annotation

**Figure 2:**
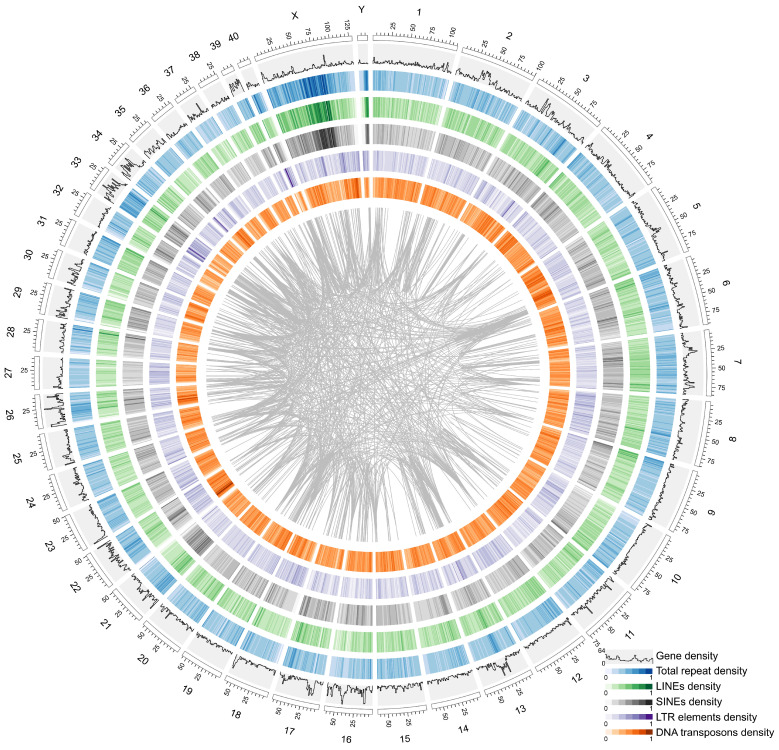
Chromosomal distribution of genes, repetitive elements, and segmental duplications in the southern white rhinoceros genome. The outer ideogram represents the assembled chromosomes. From outer to inner, the tracks indicate gene density, total repeat density, LINE density, SINE density, LTR element density, and DNA transposon density. Gray links in the center denote segmental duplications (SDs) between homologous genomic regions. Together, these tracks illustrate the uneven distribution of genes, repeat subclasses, and duplicated segments across the genome.

We next characterized the major genomic components of the *C. s. simum* assembly. Repetitive elements occupied 1,132,544,074 bp, representing 45.65% of the assembled genome (Table 2). The transposable element composition is heavily dominated by long interspersed nuclear elements (LINEs; 18.87%) and short interspersed nuclear elements (SINEs; 13.55%), alongside smaller fractions of long terminal repeats (LTRs; 7.83%), DNA transposons (4.10%) and other repeat types (1.30%). We identified 100.68 Mb of segmental duplications (SDs) distributed genome-wide with local clustering on several chromosomes (Figure 2). The Circos plot illustrates SDs interspersed across chromosomes, overlapping with gene-dense regions and major repeat classes (LINEs, SINEs, LTRs, DNA transposons), indicating their integral role in genome architecture.

Protein-coding gene annotation identified 22,593 genes, of which 21,945 genes (97.13%) were functionally annotated in at least one public database. Specifically, robust homology support was obtained from EggNOG (96.22%), TrEMBL (96.72%), Swiss-Prot (94.95%), and InterPro (89.96%), complemented by pathway and ontology mapping via KEGG (74.87%) and GO (66.05%) (Table 3). These data suggest a highly complete and functionally supported gene set. We further examined 648 protein-coding genes that lacked significant functional annotation by summarizing their coding length, exon structure, repeat overlap, and conservation in another rhinoceros genome (Table S5). Most of these genes encoded short proteins, with a median coding-sequence length of 186 bp and a median predicted protein length of 61 amino acids. Nevertheless, 619 genes showed detectable support in another rhinoceros genome, suggesting that most were not individual-specific annotation artifacts. A subset of 212 genes overlapped repetitive elements over more than 50% of their gene bodies and were therefore interpreted cautiously as repeat-associated models.

### 3.3. Structural Rearrangements of the MHC Class II Region Across Perissodactyla

**Figure 3:**
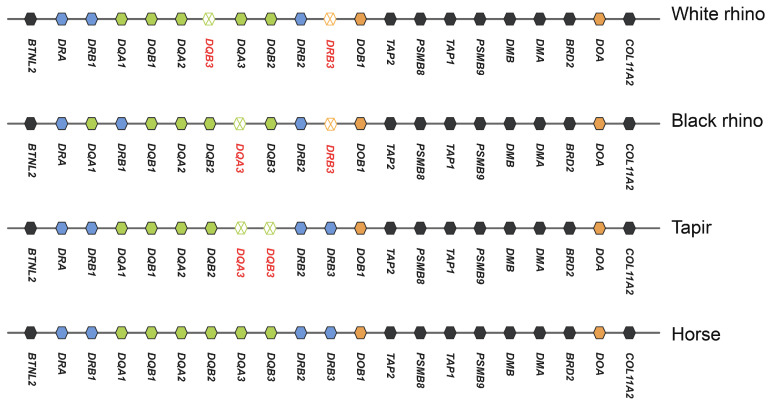
Comparative organization of the major histocompatibility complex class II (MHC-II) region in the southern white rhinoceros (*C. s. simum*), black rhinoceros (*Diceros bicornis*), tapir (*Tapirus terrestris*), and horse (*Equus caballus*). The white rhinoceros configuration shown here is shared by *C. s. cottoni*. Annotated genes are shown across the MHC-II region. Genes are shown in genomic order from *BTNL2* to *COL11A2*, with corresponding orthologs indicated by consistent colors across species. DR genes are shown in blue, DQ genes in green, DO genes in orange, DP genes in purple, and other highly conserved genes in black. Red labels indicate loci present in the horse configuration but not detected in the compared lineage at the corresponding position. Note that the nomenclature does not reflect orthology.

We assessed local assembly and annotation accuracy by characterizing the major histocompatibility complex class II (MHC-II) region, a functionally important and structurally informative immune locus. Both the southern and northern white rhinoceros genomes contained a coherent MHC-II gene block with identical gene composition and order, indicating good conservation within *Ceratotherium simum* (Figure 3).

However, broader comparisons across Perissodactyla (with black rhinoceros, tapir, and horse) revealed dynamic evolution within this locus. In particular, the flanking and framework portion of the region, including *BTNL2*, *DRA*, *DOB1*, and the segment extending from *TAP2* to *COL11A2*, showed a largely conserved organization across the compared perissodactyls. In contrast, the central DR/DQ subregion has undergone extensive, lineage-specific copy number variation and spatial reorganization. Relative to the horse genome configuration (three *DQA*, three *DQB*, and three *DRB* loci), the white rhinoceros retains three *DQA*, two *DQB*, and two *DRB* loci. The black rhinoceros exhibited a distinct copy-number configuration (retaining two *DQA*, three *DQB*, and two *DRB*) accompanied by unique spatial reordering within the left portion of the subregion. The tapir displays yet another distinct configuration (two *DQA*, two *DQB*, three *DRB*). These independent structural trajectories underscore the DR/DQ subregion as a dynamic hotspot for evolutionary contraction and rearrangement during perissodactyl divergence. Given that MHC class II genes frequently undergo lineage-specific duplications and losses, the gene nomenclature used here denotes locus-level assignments rather than strict one-to-one orthologous relationships. Consequently, gene-count comparisons across species reflect compositional differences within the annotated MHC region rather than direct ortholog counts.

Overall, these results indicate that the southern white rhinoceros genome contains a well-annotated protein-coding gene set and that the current assembly helps resolve a complex immune gene locus, thereby providing a basis for downstream functional and comparative genomic analyses.

### 3.4. White Rhinoceros Subspecies Exhibit Chromosome-Scale Synteny with Localized Structural Variation

**Figure 4:**
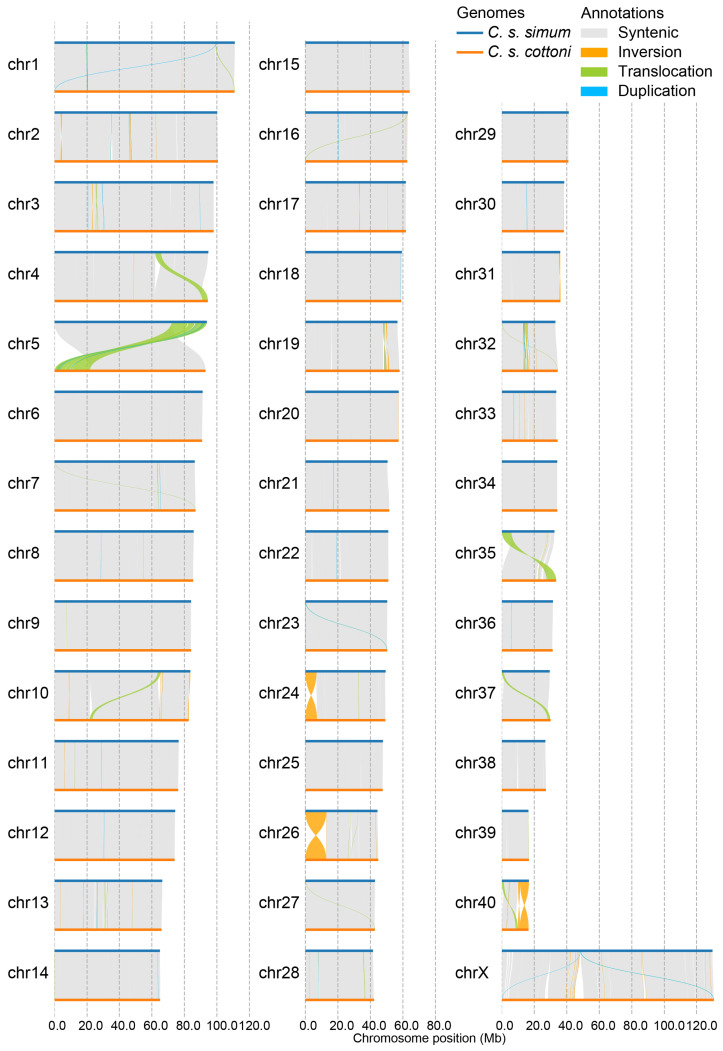
Genome-wide structural comparison between *C. s. simum* and *C. s. cottoni* based on SyRI analysis. Blue and orange horizontal lines represent the two chromosome-level assemblies, and colored links indicate syntenic regions and structural rearrangements, including inversions, translocations, and duplications.

Whole-genome comparison between the *C. s. simum* and *C. s. cottoni* showed extensive chromosome-scale collinearity (Figure 4). We resolved 2.36 Gb of syntenic sequence, accounting for 95.81% of the *C. s. simum* chromosome assembly. This near-complete structural conservation indicates that subspecific divergence in white rhinoceroses has proceeded without large-scale karyotypic reshuffling.

Instead, genomic differentiation is mainly associated with localized structural rearrangements. Structural variant (SV) profiling identified 111 inversions (spanning 33.48 Mb) and 497 translocations (spanning 36.48 Mb), alongside 23.71 Mb of insertion/deletion events (Table 4). By intersecting these rearranged regions with the southern white rhinoceros gene annotation, we identified 835 protein-coding genes affected by large SVs (≥10 kb) within their coding sequences (Table S6 and Figure S2). Pathway analysis of these genes identified an overrepresentation of genes governing cellular architecture (cytoskeletal organization, membrane dynamics), chromatin remodeling, and DNA metabolic activities. In addition, several enriched categories were linked to autophagy and immune-associated regulation, including regulation of interferon-beta production. These results suggest that lineage-specific structural variants may contribute to fine-scale regulatory or functional divergence in intracellular organization and immune responsiveness between the two white rhinoceros subspecies.

### 3.5. Phylogenetic Relationship of the White Rhinoceros

**Figure 5:**
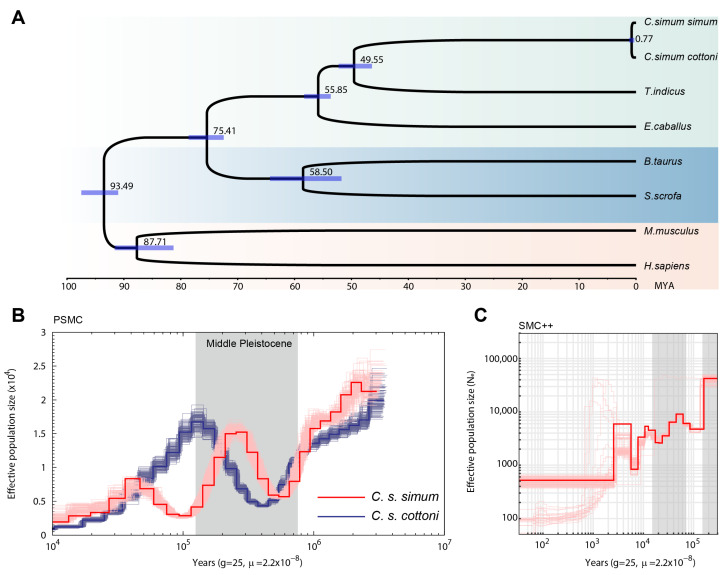
Phylogenetic relationship and historical demographic dynamics of white rhinoceroses. (**A**) Time-calibrated phylogenetic tree reconstructed from 11,831 single-copy orthologous protein-coding genes. (**B**) PSMC-inferred changes in effective population size (*N*e) for the southern white rhinoceros (red) and northern white rhinoceros (blue). Solid lines represent inferred demographic trajectories, and pale lines indicate bootstrap replicates. (**C**) SMC++-inferred demographic history of the southern white rhinoceros based on five individuals. The solid red line indicates the inferred effective population size trajectory, and pale red lines represent bootstrap replicates. Time scaling for demographic inference used a generation time of 25 years and a neutral mutation rate of 2.2 × 10^−8^ substitutions per site per generation.

To provide an evolutionary framework for the comparative genomic analyses, we reconstructed a time-calibrated phylogeny using 11,831 single-copy orthologous protein-coding genes from representative mammals, including the southern white rhinoceros, northern white rhinoceros, tapir, horse, cattle, pig, mouse, and human. The inferred topology placed the southern and northern white rhinoceroses as sister lineages, with an estimated divergence time of approximately 0.77 million years ago (Figure 5A). This recent split within *Ceratotherium simum* is consistent with previous genomic and population genetic studies showing that the two white rhinoceros subspecies are closely related but genetically distinguishable lineages. Overall, this topology agrees with previous phylogenomic analyses of Rhinocerotidae and broader mammalian phylogeny, supporting the reliability of the ortholog set used in this study.

### 3.6. Asynchronous Historical Demographic Fluctuations for White Rhinoceros Subspecies

We inferred historical changes in effective population size using PSMC [53] and SMC++ [54]. For the northern white rhinoceros individual, we called 1,913,176 heterozygous SNPs and 1,095,433 homozygous alternate SNPs, corresponding to 3,008,609 non-reference SNPs and a heterozygosity estimate of 8.36 × 10^−4^. In comparison, the southern white rhinoceros individual retained 1,459,523 heterozygous SNPs and 13,197 homozygous alternate SNPs, corresponding to 1,472,720 non-reference SNPs and a heterozygosity estimate of 6.30 × 10^−4^. The higher heterozygosity observed in the northern white rhinoceros is consistent with previous genomic studies reporting higher genome-wide diversity in northern than in southern white rhinoceroses.

To assess the effect of PSMC interval specification, we compared the refined pattern -p “1+1+1+1+25*2+4+6” with the conventional pattern -p “4+25*2+4+6”. The conventional pattern produced a pronounced and bootstrap-unstable peak in the most recent time interval, whereas this peak was substantially reduced when the first time window was split under the refined pattern (Figure S3). Coalescent demographic analyses indicated that despite their close phylogenetic affinity, the southern and northern white rhinoceroses experienced asynchronous population dynamics during the Pleistocene (Figure 5B). Rather than exhibiting long-term stability, both lineages underwent intense, independent cycles of expansion and contraction. Specifically, both *C. s. simum* and *C. s. cottoni* showed relatively large effective population sizes (*N*e) in the deeper past, followed by a marked reduction during the Middle Pleistocene. After this decline, the two subspecies followed different trajectories. In *C. s. simum*, the effective population size subsequently increased and formed a pronounced mid- to late-Pleistocene peak at approximately 2–3 × 10^5^ years ago. After this peak, the southern white rhinoceros showed a decline toward approximately 1 × 10^5^ years ago, followed by a smaller increase around 4–5 × 10^4^ years ago and a subsequent reduction toward the recent period. In contrast, *C. s. cottoni* showed a more continuous increase after the mid-Pleistocene decline, reaching a local peak around 1.2 × 10^5^ years ago before decreasing toward the recent period.

For the southern white rhinoceros, SMC++ also supported substantial demographic fluctuations through time while providing improved resolution in the more recent interval (Figure 5C). Although the fine-scale shape of the SMC++ trajectory differed from that of PSMC, both approaches consistently indicated that *C. s. simum* experienced repeated long-term changes in effective population size, with reduced recent Ne relative to deeper historical intervals. This asynchronous pattern is consistent with previous genomic studies showing that the northern and southern white rhinoceroses are closely related but genetically distinct populations with non-identical demographic histories [7,9].

## 4. Discussion

In this study, we present a chromosome-level reference genome for the southern white rhinoceros and use it to examine genome architecture, immune-region organization, structural variation, and historical demography. Compared with the previously fragmented reference, this assembly provides improved long-range genomic context for analyses of repeat-rich regions, segmental duplications, and major immune loci, which are difficult to resolve in short-read or poorly scaffolded genomes.

The MHC class II region illustrates how a high-quality assembly can clarify locus-level evolutionary patterns. Between the southern and northern white rhinoceros, this region shows conserved gene order and content, supporting the consistency of local assembly and annotation. Across Perissodactyla, however, the comparison reveals a more dynamic pattern. The flanking framework region, particularly the interval from *TAP2* to *COL11A2*, is largely conserved, whereas the central DR/DQ subregion shows lineage-specific differences in copy number and arrangement. This contrast is consistent with the broader view that mammalian MHC-II regions combine a conserved structural backbone with localized immune-gene plasticity [17,43].

At the genome-wide scale, the comparison between the two white rhinoceros subspecies reveals broad chromosomal synteny, indicating that their divergence has not involved extensive karyotypic reshuffling. Instead, structural differentiation is concentrated in localized inversions, translocations, and insertion/deletion events. Genes associated with these large structural variants are enriched for functions related to cytoskeletal organization, membrane dynamics, chromatin remodeling, DNA metabolism, autophagy, and immune regulation, including regulation of interferon-beta production. These pathways point to candidate biological axes that may be relevant to functional divergence or demographic history, but they should not be interpreted as direct evidence of adaptation without population-level and functional validation.

These structural variants should also be interpreted in the context of severe demographic bottlenecks in white rhinoceros. Reduced effective population size can weaken purifying selection and increase the probability that mildly deleterious or neutral variants persist through genetic drift [12]. Accordingly, the structural differences observed between the two reference genomes may reflect a mixture of demographic history, individual polymorphism, assembly effects, and possible functional divergence. The present data therefore help prioritize genomic regions for long-term monitoring, but they cannot determine whether specific variants are fixed within either subspecies or whether they have measurable phenotypic consequences. Resolving these alternatives will require additional individuals, long-read validation across samples, and functional or transcriptomic evidence.

Demographic reconstructions provide a temporal framework for interpreting these genomic patterns. Both subspecies show reduced recent effective population sizes relative to deeper Pleistocene estimates, but their inferred trajectories are not identical. This pattern is compatible with geographically separated histories and with repeated Pleistocene shifts in African savanna and woodland habitats [60,61], which may have altered the range and connectivity of grazing megaherbivore populations [9,61]. Nevertheless, these reconstructions should be viewed as coalescent-based hypotheses rather than direct records of census size. The comparison is also methodologically uneven: SMC++ could be applied only to southern white rhinoceros data, whereas inference for the northern white rhinoceros relies on a single genome. The apparent demographic divergence should therefore be treated as a plausible model that requires testing with broader northern white rhinoceros genomic data, should such samples become available.

For conservation genomics, the main value of this assembly lies in the improved resolution it provides for population monitoring and management. A chromosome-level reference enables more reliable profiling of single-nucleotide variants, structural variants, immune-locus diversity, and potentially deleterious coding variation. For the southern white rhinoceros, these resources can support genetic monitoring, relatedness management, and the identification of individuals that retain underrepresented genomic diversity. For the functionally extinct northern white rhinoceros, the comparative framework can help evaluate cryopreserved cell lines, guide assisted-reproduction programs, and monitor northern-specific variation during genomic rescue [11]. More broadly, chromosome-level genome resources can support future integration of conservation genomics with pedigree management, assisted reproduction, and genomic-prediction approaches increasingly used in animal breeding and genetic resource management [13,14,15,62]. These applications should proceed with clear limitations in mind: the new assembly represents a single southern individual, and comparisons with the northern white rhinoceros remain anchored to a public reference that may contain local assembly errors or unresolved repetitive regions.

## 5. Conclusions

In conclusion, we generated a high-quality chromosome-level genome assembly for the southern white rhinoceros and combined it with comprehensive annotation of repetitive elements, protein-coding genes, and segmental duplications. The distinct demographic histories and lineage-specific genomic structures documented here suggest that the two subspecies may benefit from subspecies-specific genetic monitoring and conservation planning. By characterizing the structural, functional, and demographic features of *C. s. simum* in detail, this assembly provides a useful foundation for targeted conservation efforts, including genetic monitoring, management of structural diversity, selection of genetically informative individuals or cryopreserved cell lines, and future assisted-reproductive and genomic-rescue programs aimed at supporting the long-term viability of rhinoceroses and other threatened large mammals.

## Figures and Tables

**Table 1:** Statistics of the assembled southern white rhinoceros genome.

Category	Data
Contig N50 (Mb)	42.06
Number of contigs	297
Total length of contigs (Gb)	2.48
Scaffold N50 (Mb)	66.38
Number of chromosomes	40 + XY
Total size of assembled chromosomes (Gb)	2.46
Complete BUSCOs (Benchmarking Universal Single-Copy Orthologs, %)	99.72
QV (consensus quality value)	43.52

**Table 2:** Composition of repetitive elements in the final genome assembly.

Type	Repeat Size (bp)	% of Genome
SINEs	336,129,842	13.55
LINEs	468,291,866	18.87
LTR	194,293,900	7.83
DNA	101,694,894	4.10
Other	32,133,572	1.30
Total	1,132,544,074	45.65

**Table 3:** Functional annotation of predicted protein-coding genes.

Database	Annotated	Annotation Rate (%)
GO	14,923	66.05
InterPro	20,324	89.96
KEGG	16,916	74.87
Swiss-Prot	21,453	94.95
TrEMBL	21,852	96.72
EggNOG	21,741	96.22
Total	21,945	97.13

**Table 4:** Genome-wide structural and sequence variation between the *C. s. simum* (reference) and *C. s. cottoni* (query) identified by SyRI.

Variation Type	Count	Length in Reference (bp)	Length in Query (bp)	Genes
Syntenic regions	786	2,356,932,239	2,345,017,488	-
Insertions	567,180	-	7,037,746	-
Deletions	327,567	16,673,740	-	21
Inversions	111	33,480,367	33,724,078	703
Translocations	497	36,485,447	36,359,478	98
Duplications (reference)	184	2,927,857	-	13
Duplications (query)	707	-	2,308,959	-

## Data Availability

The genome assembly of *C. s. simum* has been deposited in the Science Data Bank at https://doi.org/10.57760/sciencedb.33936 and has also been released in NCBI GenBank under BioProject PRJNA1418492, with the assembly accession GCA_057802395.1. The raw sequencing datasets generated in this study have been deposited in the NCBI SRA database under the same BioProject accession.

## Supplementary Materials

The following supporting information can be downloaded at: https://www.mdpi.com/article/10.3390/biology15120924/s1, Figure S1: ONT read-based validation of representative large structural variants; Figure S2: Functional enrichment of genes associated with SyRI-identified structural variants between the southern and northern white rhinoceroses; Figure S3: Sensitivity of PSMC trajectories to alternative interval patterns. Table S1: Structural variant loci with raw ONT read support at both reference-side breakpoints. Table S2: Public SRA datasets of southern white rhinoceros individuals used for SMC++ analysis; Table S3: Summary of sequencing results in this study; Table S4: Genome quality comparison of available *C. s. simum* genome assemblies. Table S5: Protein-coding genes lacking functional annotation in the southern white rhinoceros genome. Table S6: Functional enrichment of SV-associated genes identified from the southern white rhinoceros genome in this study.
