# Supplementary material for: Chromosome-Scale Genome Architecture and Historical Demography of the Southern White Rhinoceros
Source: Biology (Basel). 2026 Jun 12;15(12):924. doi: 10.3390/biology15120924 (PMC13296254; doi:10.3390/biology15120924)
Supplement: Supplementary file 1 [file biology-15-00924-s001.zip › Supplementary_file1.pdf]

Table S1. Structural variant loci with raw ONT read support at both reference-side breakpoints. For each structural variant (SV) locus, support was defined as raw ONT reads that fully spanned both breakpoints of the locus. The table reports the number of SV loci retained when both breakpoints were supported by at least the indicated number of raw ONT reads.

**Minimum ONT reads support at both breakpoints of an SV locus**	**Number of SVs identified**
>20 reads	408,978
>10 reads	857,369
>5 reads	891,111
>0 reads	896,246

Table S2. Public SRA datasets of southern white rhinoceros individuals used for SMC++ analysis.

SRA	Sample	depth
SRR387388	KB13650	16.47×
SRR5852740	KB7062	15.62×
SRR5852739	KB5892	15.24×
SRR5852738	KB6974	14.09×

Table S3. Summary of sequencing results in this study.

Species	Libraries	Total data (Gb)	Depth (×)
Ceratotherium simum simum	Nanopore	195.6	81.5
	Illumina	151.8	63.25
	Hi-C	34.3	14.29

Table S4. Genome quality comparison of all available *C.s.simum* genomes.

	GCA_000283155.1	This study
Genome size	2.46 Gb	2.48 Gb
Contig N50	93 Kb	42.06 Mb
Number of contigs	57,823	297
Number of scaffolds	3,086	76
Scaffold N50	26.3 Mb	66.38 Mb

Table S6. Functional enrichment of SV-associated genes identified from the southern white rhinoceros genome in this study.

Category	Term	Description	LogP	Log(q-value)
GO Biological Processes	GO:0030036	actin cytoskeleton organization	-5.970	-1.895
GO Biological Processes	GO:0042593	glucose homeostasis	-5.785	-1.895
GO Biological Processes	GO:0051668	localization within membrane	-5.443	-1.761
GO Biological Processes	GO:0006338	chromatin remodeling	-5.400	-1.761
GO Biological Processes	GO:0006914	autophagy	-5.218	-1.748
GO Cellular Components	GO:0070603	SWI/SNF superfamily-type complex	-5.182	-1.748
GO Biological Processes	GO:0072594	establishment of protein localization to organelle	-5.110	-1.745
GO Biological Processes	GO:0010256	endomembrane system organization	-4.842	-1.583
GO Molecular Functions	GO:0140640	catalytic activity, acting on a nucleic acid	-4.667	-1.57
GO Biological Processes	GO:0006259	DNA metabolic process	-4.633	-1.57
GO Cellular Components	GO:0098793	presynapse	-4.626	-1.57
GO Cellular Components	GO:0031252	cell leading edge	-4.619	-1.57
GO Cellular Components	GO:0019866	organelle inner membrane	-4.602	-1.57
KEGG Pathway	hsa05132	Salmonella infection	-4.586	-1.57
GO Biological Processes	GO:0032648	regulation of interferon-beta production	-4.478	-1.502
GO Biological Processes	GO:0031399	regulation of protein modification process	-4.439	-1.481
GO Biological Processes	GO:0098657	import into cell	-4.365	-1.445
GO Biological Processes	GO:0010638	positive regulation of organelle organization	-4.335	-1.444
GO Molecular Functions	GO:0030275	LRR domain binding	-4.300	-1.442

GO Biological Processes	GO:0009896	positive regulation of catabolic process	-4.226	-1.42

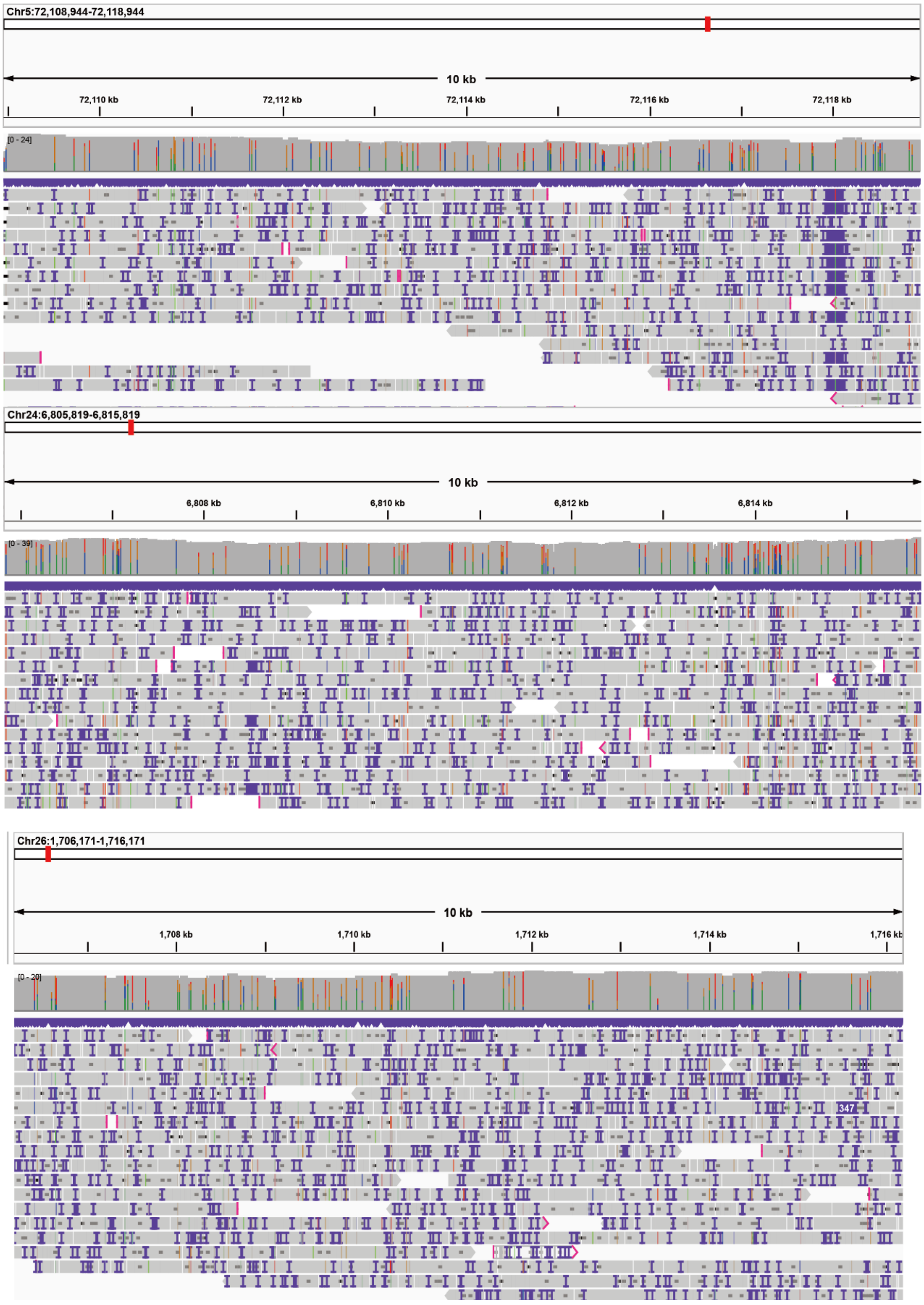

Figure S1: ONT read-based validation of representative large structural variants. IGV views of ONT long-read alignments at three representative large SV loci in the southern white rhinoceros genome of this study (Chr5:72,108,944–72,118,944; Chr24:6,805,819–6,815,819; Chr26:1,706,171–1,716,171). The consistent alignment patterns observed across multiple long reads support the authenticity of these large SVs identified from assembly-based genome comparison.

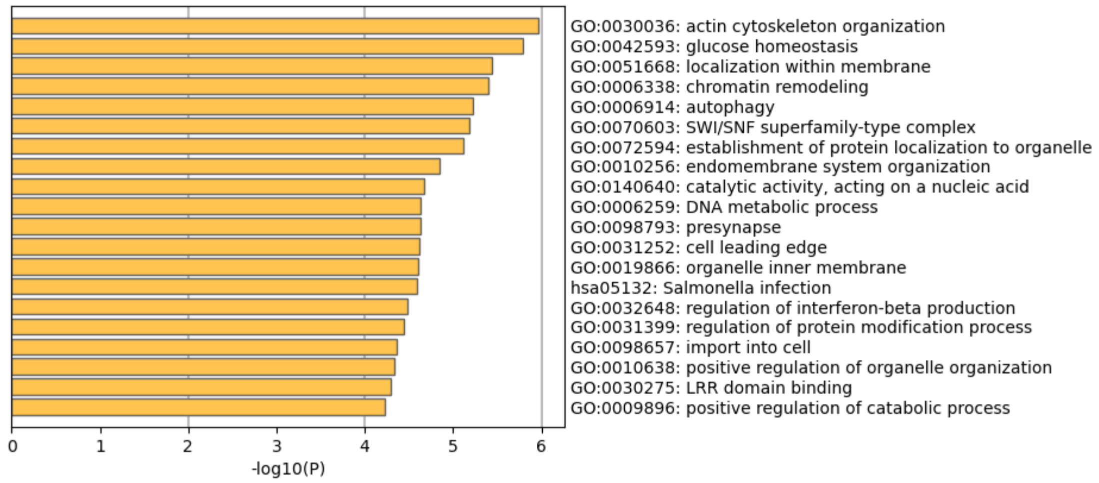

Figure S2: Functional enrichment of SV-associated genes identified from the southern white rhinoceros genome in this study.

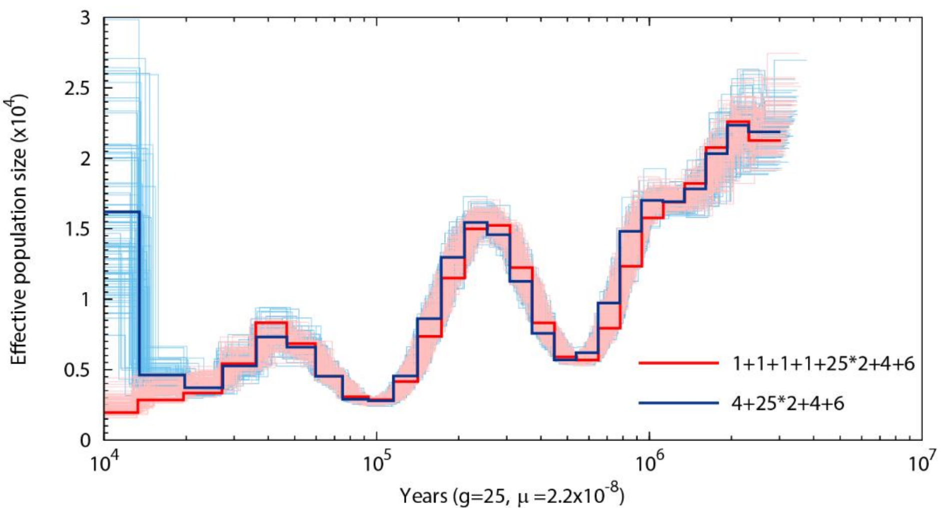

Figure S3. Sensitivity of PSMC trajectories to alternative interval patterns. PSMC-inferred demographic trajectories were compared between the refined interval pattern -p "1+1+1+1+25*2+4+6" and the conventional interval pattern -p "4+25*2+4+6". The conventional pattern produced a pronounced and bootstrap-unstable peak in the most recent time interval, whereas this recent peak was substantially reduced when the first time window was split under the refined pattern. In contrast, the broader Pleistocene-scale trajectory was largely consistent between the two parameter settings.
